# Ethical and Legal Aspects of Telerehabilitation in Saudi Arabia

**DOI:** 10.5195/ijt.2023.6569

**Published:** 2023-12-12

**Authors:** Ahmed Mushabbab Alhabter, Ahmad Zaheer Qureshi, Sami Ullah

**Affiliations:** 1 Medical Affairs, King Faisal Medical City Abha, Saudi Arabia, Mailing Address: King Faisal Medical City, Al Andalus Abha 62521 Saudi Arabia; 2 Department of Physical Medicine & Rehabilitation, King Fahad Medical City Riyadh, Saudi Arabia, Mailing Address: Rehabilitation Hospital, King Fahad Medical City, Sulaymania Riyadh 11525 Saudi Arabia

**Keywords:** Ethics, Legal, Saudi Arabia, Telemedicine, Telerehabilitation

## Abstract

Organizations have their own policies and procedures to govern operational aspects of health care facilities. With the advent of telemedicine, there has been a growing trend in providing telehealth practices without formally exploring the ethical and legislative aspects. The potential use of electronic and digital services in telerehabilitation can influence various ethical and legal factors, such as confidentiality, consent. and negligence. Thus, establishing clear strategies in this regard is necessary. Ethical and legal aspects of healthcare are influenced by cultural, religious, and legislative rulings of a state. At the same time, the multidimensional scope of rehabilitation in a health system has its own challenges. This narrative review intends to highlight the importance of incorporating the ethical and legislative framework in the telerehabilitation process in Saudi Arabia. A summary of various aspects in-line with unique local attributes is included, which can also help to facilitate regional telerehabilitation services in the Arab World.

Telerehabilitation operates differently than other fields of telemedicine. This is mainly due to the holistic nature of rehabilitation care, which not only involves the biological aspects of disease but also addresses the psychological and social aspects of patients (Frontera et al., 2019). The scope of care extends beyond the patient and addresses the challenges faced in relation to the family, environment, and society at large. Vocational and social empowerment is often the main goal of rehabilitation for many individuals with disabilities (Kamper et al., 2014; National Task Force on Workforce Development for People with Disabilities, 2016). Hence, rehabilitation plans are not only individualized to meet the needs of care for the patient but also considerably dependent on cultural and societal norms, which in turn are strongly linked with the health care system, legislation, and disability laws in a country. Due to the complex variation in rehabilitation practices around the world, there is a dire need for focusing on practice standardization in telerehabilitation locally, as the data on ethical and legal aspects in the country are lacking. (Clinical Governance Directorate, 2020; El Kheir et al., 2022; Qureshi et al., 2021). Considerable care must be taken when delivering remote health care through telemedicine, especially in situations where a medico-legal framework is lacking, and providers and patients are not well versed with the limitations of telemedicine. This is of particular importance in regions where religious and cultural implications in health system delivery carry high value (Koenig et al., 2001; Skotnicki, 1996; Yamey & Greenwood, 2004). Clear guidance towards ethical and legal consideration for telerehabilitation in Saudi Arabia and Middle East is lacking (Almubark et al., 2022; Alqahtani et al., 2021). In many situations, perceptions of disability and unrealistic expectations of recovery stand out as the most challenging issues in the rehabilitation process (Kane, 1997; Wilde et al., 1993; Wressle et al., 2006). Common medical ethics dilemmas faced in Saudi Arabia include end-of-life care and patient rights, which comprise autonomy, informed consent, confidentiality, reproductive ethics, and equity of resources (Alnamlah et al., 2022).

The provision of healthcare services through telerehabilitation should not entail any exemption from adhering to ethical and legal principles. Telerehabilitation encompasses multiple disciplines of rehabilitation working coherently to achieve certain goals. The interdisciplinary nature of rehabilitation renders the need for close communication among all team members as well as patients and their families. This model is challenging to implement in remote care, as it is very important to ensure continued communication among rehabilitation team members beyond telerehabilitation sessions with patients. Rehabilitation team members typically include rehabilitation physicians, rehabilitation nurses, physical therapists, occupational therapists, speech therapists, swallowing therapists, orthotists, assistive technologists, case coordinators, social workers, recreational and art therapists, spiritual or religious service providers, dieticians, mental health providers and, in certain health systems, music and pet therapists. Given its unique setup, telerehabilitation has a different perspective that requires special consideration in the application of an interdisciplinary model of care. In this article, some of the important differences in ethical and legal applications between conventional models of rehabilitation care and telerehabilitation are highlighted.

One hypothetical example is mentioned below to highlight the challenges that can potentially be faced in telerehabilitation (Table 1). The scenario below is a combination of different situations that routinely appear in local rehabilitation encounters, both in inpatient and outpatient settings. The discussion elaborated later in the article does not specifically relate to the example below but can be reviewed in the context of this case.

**Table 1.** Ethical Challenges of Telerehabilitation in Context of Sample Case

Remote area with limited access to specialized care and technology offering suboptimal assessment via telerehabilitation. Poor socioeconomics and complex family dynamics - many family members attending the telerehabilitation session; multiple opinions; peer pressure among family members; difficult to satisfy and address concerns of all family members attending the online session; privacy issues. Chronic severe injury, poor prognosis, and multiple complications - requires complex information to be addressed via remote communication. Influence of tribal traditions and use of cultural alternative treatments with conviction are difficult to address via online meetings, especially when such treatments are carried out by recommendations of senior family members. Detailed examination could be limited in the telerehabilitation session as per preference of the family, who do not wish the patient to be exposed or examined in front of the camera. High and unrealistic family expectations, influenced by belief systems - difficult to address via discussion online. The possibility of medico-legal consequences as the family wants to record and produce discussion in court for legal settlements which they do not wish to disclose. The family is concerned about the past medical treatment during hospitalization and wants the opinion of the telerehabilitation team. Lack of specialized services at local facilities - continuity of care after the telerehabilitation session could be challenging.

## Disclaimer

The following case scenario is intended to give a contextual perspective for subsequent sections. The circumstances of the case scenario are not considered typical in our country. Some issues raised in the scenario are not even considered normal in our society. Nonetheless, it is not uncommon for healthcare providers in Saudi Arabia to encounter one or more of the ethical and legal issues addressed. Despite that, some people might find this scenario offensive. Thus, discretion is advised for readers.

## Sample Case Scenario

Sequelae of traumatic brain injury (TBI): A 24-year-old married female from a *remote area of* the country coming from a *poor socioeconomic background* had a TBI 2 years ago. A telerehabilitation session will be carried out for the first time with her and her family remotely by a team of TBI experts from a specialized rehabilitation center while she is at a clinic of a local rehabilitation facility. The local rehabilitation facility has been involved in her care only for the last six months, as she was referred to them recently. She remained in a *coma for the first five months* after her injury and later stayed in the trauma center for one year, as the family could not provide care at home, and only bed-side rehabilitation therapies were available during her hospitalization. The local rehabilitation provider mentioned that the patient was in a *minimally conscious state, had contractures of all extremities, was malnourished, and had a sacral pressure ulcer.* She was provided with the required orthotics, equipment, and home care services alongside treatment via the available rehabilitation therapies and referrals to orthopedics and plastic surgery at the local facility. The patient is now at home, living on the ground floor of a villa owned by the husband, who has a second wife living with him on the second floor. The local rehabilitation team would like to consult TBI rehabilitation experts via telerehabilitation regarding the possibility of improving her arousal state, treating her contractures, improving her nutritional status, and evaluating a proper seating system. The primary neurosurgeon and the local rehabilitation providers have already extensively counselled the family about her poor prognosis of recovery and have suggested a treatment plan to lessen the burden of care on the family and to prevent complications of prolonged immobility. *The patient's husband and brothers are very excited to carry out the telerehabilitation session* with TBI experts from a specialized center operating under the Ministry of Health.

1. They are expecting immediate transfer and admission to the specialized TBI rehabilitation center.
2. *They are expecting the patient to return to her pre-trauma healthy state and to be “normal.”*
3. The husband wants to know *if the patient can become pregnant again after her “recovery,” and if not, he would like that to be stated clearly in a medical report*.
4. The five brothers of the patient, who all live nearby, insist on attending *the whole telerehabilitation session with consent of husband* and have asked their relative who works at the same rehabilitation facility to attend the session with them.
5. They would like to know *if they can record the session on their personal phones so it can be shared with other family members not present in the clinic, and they would like to reproduce the recording in the court for some legal settlements that they do not wish to disclose*.
6. *Family members have tried various traditional and alternative treatments and express a strong belief that the patient will have a miraculous recovery*.
7. The family would like to know *if there was a delay in referral* from the trauma team to the rehabilitation center, as they believe that this could have prevented all the complications.
8. The family clearly notes that the *therapies received by the patient are not enough* and that if the prognosis remains unchanged, the specialized rehabilitation center should clearly explain that they cannot provide service for the patient and should make a referral for rehabilitation treatment outside the country so the treatment abroad can be funded by the government.
9. *The husband does not want the patient to be observed or* exposed for examination and evaluation *via live telecommunication*. The patient lays on a stretcher wearing a traditional veil and is accompanied by a female home care nurse. The local rehabilitation team includes female therapists, a female nurse, and a male rehabilitation medicine consultant. The local rehabilitation facility does not have speech or swallowing services. The session is carried out live via the telecommunication audio-video platform installed at the two centers as a part of a telemedicine initiative by health authorities.

## Methods

This article provides a narrative review of the ethical and legal principles relevant to telerehabilitation in Saudi Arabia. We conducted a search of databases including Academic Search Premier, Ovid, PubMed, and Google Scholar. The search included both sources in English and Arabic. The keywords are categorized into one of three domains: ethical and legal principles, telemedicine and telerehabilitation, and religious and cultural attributes. Search words included *“ethics”, “legal”, “negligence”, “consent”, “telerehabilitation”, “telemedicine”*, “*Middle East*”, “*Saudi Arabia*” and “*Islam*”. We also included relevant legislation in Saudi Arabia. Qualitative and quantitative research, laws, and online resources were looked at, as the purpose of the review was to locate local data relevant to ethical and legal practices in context of telerehabilitation. Initial inclusion criteria of availability in English, local rehabilitation literature and telemedicine narrowed down the search to 1400 resources, which included journal articles, books, online reports, and governmental resources. Reference lists of retrieved studies were hand searched for other relevant citations. Articles were then narrowed to examine the ethical, legal, and religious relevance to Saudi Arabia and the Middle East. Interestingly, the number of relevant publications has increased by more than threefold since COVID-19 pandemic. After eliminating duplicates, 58 resources were reviewed. We created a template based on the common headlines in this context and then summarized the review based on the national regulations as well as cultural attributes of Saudi Arabia.

### Observance of Religious and Cultural Rulings

Like other nations, Saudi Arabia has a unique culture. It also follows Islamic rulings, which are commonly referred to as the *“Islamic Sharia law.”* Notably, Islamic Sharia law can be seen as a hybrid of the common and civil law systems (Powell & Mitchell, 2007). It occasionally relies on precedents but sometimes depends on written legislation. The supporting evidence, or “*daleel”*, is a crucial source of rulings in Islam. Based on its authenticity, it can supersede both the precedents and the written legislations. Therefore, providers of healthcare must consider these rules and norms. Among the common Islamic rulings that must be considered are those on the exposure of private body parts (*Awra*), treatment of patients of different sexes, female companions (*Mahram*) and end-of-life issues. One proposed approach to the resolution of any ethical or legal dilemma from the Islamic perspective is to look for the supporting evidence (*daleel*) or any precedent case to reach a decision, or *hukm* (Sachedina, 2009). The Islamic etiquettes of visiting patients can be quite different from regular practice (Wani, 2019). These etiquettes and cultural habits must be respected, especially by the home-based telerehabilitation team.

Given the infinite scenarios that the telerehabilitation team can come across, it is not feasible that any policy or procedure could cover them all. Therefore, it is critical to establish clear guidance or protocols. It is recommended to add a general statement in the program policies that can help the team look for a proper resolution of moral or legal aspects of care.

### Codes of Ethics

All healthcare professionals, including clinicians and researchers, must apply basic ethical principles. This includes modern bioethical principles, including autonomy, beneficence, non-maleficence, justice, veracity, fidelity, and privacy (Phalen, 2017). It is recommended, however, that breaking bad news be conducted in person without physical barriers (Miller, 2003). Breaking bad news is one of the key interventions in rehabilitation practice, as it refers to disclosure of unfavorable prognosis regarding disability or any news that drastically and negatively alters the patient's or relatives' view regarding the patient's future (Narayanan et al., 2010). This is of particular significance when patients or families are in denial or are unaware of the nature of the disability, as there is always a concern of eliciting a negative reaction from the patient or his or her relatives. Hence, telerehabilitation might not be the best platform for breaking bad news.

Healthcare practitioners in the telerehabilitation model and community health care providers have more access to patients' private and home environments. Thus, they might encounter unlawful practices, including criminal offenses. For example, substance use is considered a public health problem in Saudi Arabia (Bassiony, 2013). There is a chance that it may be encountered by health care providers at some point. This brings attention to reporting protocols of such incidents. It is therefore crucial to follow the relevant rules, such as the Saudi regulations of narcotic drugs and psychotropic substances or their equivalent (Ministry of Health, 2019; Saudi Food & Drug Authority, 2019). Most countries have similar legislation that must be considered in such incidents. The recommended action depends on whether the incident involved suspected use, possession, or dealing. It is also important to detect any potential impact on patient health and well-being. Similarly, during telerehabilitation encounters, handling the suspected abuse of individuals with disabilities in the form of assault or neglect can be a challenging situation. Each organization must consider these factors in its policies and procedures for telerehabilitation services. Telerehabilitation team members must act as advocates of their patients and consider reporting such suspicions without jeopardizing their own safety. Thus, proper orientation and training on relevant policies as well as responses during such situations can be seen as a prerequisite to starting telerehabilitation services.

### Codes of Conduct and Therapeutic Relationships

It is common sense that no treatment whatsoever can be provided without obtaining the required consent. Confirming the patient's identity is essential for all services, including the approval of treatments. This is also applicable to substitute decision makers such as guardians. The process of conducting telerehabilitation must be clear in policy documents. Relevant legislation and guidelines such as the Saudi Guidelines for Informed Consent must be considered (Ministry of Health, 2019). As per the British Medical Association Ethics Department, professionals who prescribe the treatment are responsible for informing patients (Sommerville, 2013). Agreeing to telerehabilitation care in general does not necessarily indicate consent to all intended treatments. Nevertheless, signing an informed consent implies consenting to risk that is impossible to anticipate given that new technologies may involve new kinds of risks (Kaplan & Litewka, 2008). It seems ethical, however, that those giving consent should also be aware of this. Many factors impact the process of obtaining the required consent. For example, age, cognitive ability, and mental health status are important determinants of the patient's ability to give consent. The patients' capacity will be discussed later. Informed consent should also include discussions about possible threats to privacy and confidentiality of medical information in this model of care (Bauer, 2001). These principles must also be applied to conducting research.

Unlike rehabilitation in hospital environments, telerehabilitation might place healthcare providers in challenging situations. Obtaining consent for children and young adults can be problematic. Defining the age of maturity and the need for guardianship is an essential component of the policies and procedures of telerehabilitation. Similarly, many patients with acquired brain injury may have receptive or global aphasia or severe cognitive impairment, raising concerns regarding the competency and capacity of the patient. In a telerehabilitation setting, these aspects ideally need to be addressed prior to telemedicine sessions, possibly by the primary treating physician. Not only are the disclosure of information, next of kin, advanced health care directives, and health care power of attorney poorly understood by the general public, but health care providers also find it difficult to strictly comply with these formalities. This may be largely due to the strong cultural propensity for one key family member to be nominated as the most responsible family member to coordinate the care of the patient by a general consensus among the family members without any official validation. In contrast, spouses may not be directly involved in the direct care of a patient who is not competent; rather, many family members interact with the treatment team to come up with a shared decision. On the one hand, although a large family size may seem reassuring to ensure continuity of care, it becomes challenging to handle situations if differences among family members arise. Relatives prefer to share the burden of care and rotate during clinic visits or as caregivers during admission, offering more challenges to achieving a sustainable continuum of care. This can be even more challenging in telerehabilitation settings, and members of the treatment team may have to repeat whole discussions with different family members in different sessions. On many occasions, the person attending the telerehabilitation session will not be the one providing care for the patient at home, and there is a strong possibility that the same person may not come to future appointments despite clear instructions to do so.

The safety of decisions made through telemedicine can be comparable to the safety of decisions made through in-person consultations (Hersh et al., 2002). It has been reported, however, that conducting clinical assessment via telemedicine models might not be as sensitive and has the potential to miss some cases of disease (Duncan et al., 2010; Taylor, 2005). In some instances, this can be correlated with the paucity of assessment and treatment tools that are supported by good psychometric attributes to be used in this model of care (Latifi, 2008). Telerehabilitation treatment does not focus on a single organ or organ system but rather addresses the holistic needs of patients, including the cognitive-communicative aspects, physical and psychological limitations, functional impairments, and environmental and social challenges of the patient. It also requires assessment and treatment by different rehabilitation disciplines over a course of many sessions, which can be carried out via telerehabilitation in a home setting or in coordination with local rehabilitation providers. Thus, patient safety can be jeopardized if no standardized measures are taken throughout the patients' journeys.

Despite advancements in technology, it might sometimes be impossible to determine the severity of the patient's condition through telemedicine (Clark et al., 2010). Healthcare providers must take extreme caution when prescribing treatments. This applies to all kinds of treatments, including medications, exercises, or counseling. Given the complexity of the telerehabilitation model, a more conservative prescription approach may be appropriate. It is recommended that in some cases, the first examination be conducted in person, and therapy sessions should be carried out under direct supervision by clinicians as much as possible, which can be achieved to a certain extent by offering telerehabilitation in certain domains of therapies, such as speech therapy, swallowing therapy, self-care, and training in activities of daily living.

All healthcare practitioners must maintain their professional registration and privileging according to the relevant legislation. In Saudi Arabia, the Saudi Council for Health Specialties and the Saudi Ministry of Health are the main legislatures for these issues. It is also very important that the telerehabilitation team identify the local healthcare services before commencing the required care. This includes the local family physician, the nearest emergency department and ambulance services. The use of advanced technology to detect medical emergencies, such as fall alert systems and personal emergency devices, are other options to consider. This, however, needs more planning and engagement from other stakeholders.

All devices that will be used for telerehabilitation care must be registered and licensed with the relevant party. Medical devices, including robotic treatments, must follow the Saudi Food and Drug Authority's regulations (Dickens & Cook, 2006; Alazmi & Alhamad, 2020). Other equipment, such as handheld patient devices, must follow their relevant registration and licensing rules, such as the Ministry of Commerce regulations (Ministry of Commerce, 2019). This has been flagged as a critical prerequisite for all telemedicine practices (Stanberry, 1998a). Registration and licensing are also crucial for all software applications.

The use of complementary and alternative medicine (CAM) is relatively common among Saudi patients (Alrowais & Alyousefi, 2017). Unlike patients in supervised and regulated environments such as hospitals, community patients have more liberty in using CAM treatments. Therefore, it is crucial that healthcare professionals ensure that there are no dangerous interactions between CAM therapies and allopathic treatments (Mullins-Owens, 2016).

Many regulations govern clinicians' participation in the media. In the era of social media, such participation is even more risky. Practitioners must be considerate and refrain from prohibited and unethical practices (The Saudi Commission for Health Specialties Department of Medical Education & Postgraduate Studies, 2014). A clear statement regarding this issue must be included in telerehabilitation policies.

Providers of telerehabilitation must comply with the relevant labor and occupational safety regulations. Maintaining the required accreditations, such as those issued by the Saudi Central Board for Accreditation of Healthcare Institutes (CBAHI), is also crucial. Special consideration must be given to this unique model of care, particularly home-based telerehabilitation. Hence, the extension of employees' rights, such as regarding work injuries, is essential. For example, the Saudi Social Insurance Law is flexible in defining the terms “work” and “workplace” (General Organization for Social Insurance, 2000). Therefore, defining these terms is at the employers' discretion. Clearly, this is a critical issue that must be given the attention it deserves. Other work-related rules, such as dispute, grievance, and harassment policies, must consider the context and complexity of this model. Often, telerehabilitation is provided through the collaborative work of many organizations. Therefore, it would be necessary for all participating organizations to also contribute to writing the policies and procedures.

Although most health care services provided to Saudi nationals at governmental hospitals are free of cost, there are options for private services and insurance coverage for Saudis and non-Saudi nationals. Another potential challenge is the reimbursement for telerehabilitation by insurance companies, as there is a lack of clarity on regulations regarding telemedicine (Matusitz & Breen, 2007). This is even more troublesome for services across different regions of the country, as they can lead to ambiguity in reimbursement (Stanberry, 2006). It is important to consider alternative pathways such as incorporating this model in a financially approved model or even engaging in advocacy for it to be funded.

### Advanced Care Directive

There are guidelines available regarding withdrawing or withholding lifesaving treatment from a terminally ill patient in Saudi Arabia (Hussein et al., 2015; Taskforce to prepare National policy and procedure for do-not-resuscitate (DNR) status, 2017) This is often referred to as a do not resuscitate (DNR) order or a do not treat (DNT) order. This process requires a very meticulous assessment and adherence to the relevant legislations. It is proposed that advanced care directives, including end-of-life decisions, should never be done through telerehabilitation. Nonetheless, these directives must be identified and made clear to the relevant members of the team, as they might influence their decision-making processes and ultimately their treatment plans.

### Negligence

In general, the clinicians' duty of care toward telerehabilitation patients is similar to that in conventional practice. Clear job descriptions for each team member can help establish a standard of care. Failing to meet these standards is a fundamental factor for negligence to be proven (Buchbinder et al., 2019). However, the contextual factors of telerehabilitation must be considered when judging someone's competency and compliance with the standards of care (Stanberry, 1998b).

Given the potential limitations of telerehabilitation, any practice that leads to insufficient management can be considered negligence (Stanberry, 2001). Therefore, recognizing the program's capacity and its shortcomings is of paramount importance even prior to commencement. A major concern for telerehabilitation is the identification of the legal jurisdiction and whether indemnity insurance covers the clinicians (Silverman, 2003). This must be addressed before establishing a new telerehabilitation service, particularly if there is an international partner. Rates of litigations of potential medical errors in Saudi Arabia have increased in recent years. The rates of cases reviewed by the Sharia Medical Panels in Saudi Arabia increased from 2002 clinical claims in 2012–2013 to 3043 in 2015–2016 (Alkhenizan & Shafiq, 2018). This may have an impact on the telehealth practices in general.

### Capacity

Assessing patients' capacity to make decisions is an essential step to determine their ability to give consent (Herring, 2016). This can be challenging since there are various levels of capacity (“Capacity Toolkit,” 2022). If deemed necessary, a substitute decision maker should be assigned. It is also very important to conduct a screening evaluation of patients' mental health status. This includes, but is not limited to, an evaluation of their safety. Although it can be a breach of basic human rights, treating patients against their will and disclosing confidential information might be justifiable in some cases (Kerridge et al., 2013). Thus, a prescriptive policy and procedure must be written prior to launching the service. The telerehabilitation team should know when and how to intervene. Relevant legislation, such as the Saudi Mental Health Care Law, must be considered (Ministry of Health, 2019).

### Medical Records Management

Documentation is a fundamental part of all healthcare encounters. The general principles of medical records in terms of their purpose, contents and regulations are similar between telerehabilitation and conventional healthcare organizations. Nonetheless, some aspects can be compromised in the telerehabilitation model. For example, timeliness, completeness, and information security can be difficult to sustain given the complexity of this model. Security is an ever-present concern for electronic medical records (Fremgen, 2012). This must be considered when signing any contract, as no third party, such as technical support companies, can have the right to access, retain, or disclose any confidential information.

The use of electronic modes of communications is a cornerstone of telerehabilitation. Given the potential risk of confidentiality breach, proper planning and policy making is essential even before providing the service (Lewis et al., 2012). Some telerehabilitation systems allow audio-visual recording of consultations. It has been reported that both patients and clinicians must be made aware of any recording, including audio-recording (Hill, 2006; Stanberry, 1997). It is mandatory to obtain informed consent prior to telerehabilitation encounters; however, this can be debatable, as some healthcare professionals or patients may claim their medico-legal right in recording these encounters. Therefore, when establishing telerehabilitation services, we recommend a proper legal consultation. Relevant regulations must be considered. In Saudi Arabia, for example, the Anti-Cyber Crime Law is one of many legislations that must be considered (Bureau of Experts at The Council of Ministers, 2007).

Disclosing confidential information about patients is governed by the same ethical and legal rules that are applied to healthcare organizations. It is sometimes permissible for disclosure to be performed without patient permission (Stanberry, 1997). Telerehabilitation creates more scenarios when disclosure might be necessary. For example, if a patient who resides in another city or even country is deemed unsafe, it would be acceptable to notify local authorities, including the police.

### Certificates and New Telemedicine Platforms in Saudi Arabia

It is inevitable that the telerehabilitation team will be asked to issue medical certificates such as medical reports, sick leaves, and letters regarding return to work. These documents are legally binding, and whoever issues them must be committed to their contents (Kerridge et al., 2013). Although it is feasible to issue them through a telerehabilitation model, we recommend a more conservative approach given the need to conduct comprehensive assessments. Many of the patients may have new onset illness with permanent or long-term disabilities. It is difficult to determine their ability to return to work or vocational prospects via remote assessment, and in-person services are preferable.

The COVID-19 pandemic has increased the use of digital health-care services in Saudi (Alkhalifah et al., 2022) and the Middle East (Shamiyah et al., 2023). The era of online applications has replaced paperwork and centralized many processes such as sick leave and appointment scheduling. One such application is Anat (“MOH Apps for Smartphones,” 2022). A study published in 2021 reported that six apps in total were developed prior to the COVID-19 pandemic, out of which three apps were modified to address different aspects of the pandemic (Alassaf et al., 2021). Both SEHA and Mawid included information about COVID-19 awareness. During the COVID-19 pandemic, three official apps were developed: Tawakkalna, Tetamman, and Tabaud. Additionally, 937 app offers teleconsultation as well. To address the growing needs of teleconsultations, the Ministry of Health has also launched Anat app, which is a platform to provide channels of communication between health practitioners in their various specialties.

## Conclusions

In the digital era, it is essential that health care provision be consistent with advancements in other sectors. The rehabilitation scope and the unique cultural and religious rulings of Saudi Arabia have a considerable impact on the planning and provision of telerehabilitation. Almost all aspects of health care can be influenced. Appropriate strategies need to be adapted to incorporate formal legislative measures in telerehabilitation considering ethical, religious, and sociocultural aspects of health system in Saudi Arabia.
